# *Protagonista lugubris*, a cockroach species new to China and its contribution to the revision of genus *Protagonista*, with notes on the taxonomy of Archiblattinae (Blattodea, Blattidae)

**DOI:** 10.3897/zookeys.574.7111

**Published:** 2016-03-28

**Authors:** Chenchen Wang, Zongqing Wang, Yanli Che

**Affiliations:** 1 College of Plant Protection, Southwest University, Beibei, Chongqing 400716, P. R. China

**Keywords:** Planeticinae, new record, synonym, taxonomy, *Eroblatta*

## Abstract

The blattid genus *Protagonista* Shelford, 1908, which is tentatively assigned to the subfamily Archiblattinae (= Planeticinae), is reported from China for the first time with illustrations and description of *Protagonista lugubris* Shelford, 1908. It is a wood-dwelling and potentially a wood-feeding species. The male and female genitalia of *Protagonista* are described and illustrated for the first time. The species *Protagonista pertristis* Hanitsch, 1923 is revived from the synonymy of *Protagonista lugubris*, and the remaining three nominal species that were also considered as synonyms of *Protagonista lugubris* are now recognized as synonyms of *Protagonista pertristis*. In agreement with Princis (1965), we propose that *Eroblatta* Shelford, 1910, a genus closely related to *Protagonista*, should be placed in the subfamily Archiblattinae rather than Blattinae. However, the taxonomy of Archiblattinae is problematic and awaits revision. Photos and a key to species of *Protagonista* and *Eroblatta* are provided, including photos of the holotypes of the synonymized nominal species. In addition, although Planeticinae is the senior synonym of Archiblattinae, the priority of the latter should be maintained since it is in prevailing usage based on the Article 40.2 in ICZN 4^th^ edition.

## Introduction

The cockroach subfamily Archiblattinae (= Planeticinae) belongs to the family Blattidae and is distributed in southeast Asia. Archiblattinae has a controversial taxonomic history. Planeticidae was erected by Walker (1868) based on the genus *Planetica* Saussure, 1863. This was subsequently synonymized with the genus *Archiblatta* Snellen van Vollenhoven, 1862 by Saussure (1869). It was Kirby (1904) that then established the subfamily Archiblattinae (family: Blattidae) on the basis of the genus *Archiblatta*, making Planeticidae a synonym. Meanwhile, Kirby (1904) included *Catara* Walker, 1868 in this subfamily. Handlirsch (1930) raised Archiblattinae to Archiblattidae, which was assigned to Blaberoidea by Princis (1960).

Shelford (1908, 1910) established *Protagonista* and *Eroblatta*, respectively, and placed them in the subfamily Blattinae (note all cockroaches were included in Blattidae). Princis (1965) included the four genera listed above in Archiblattidae. Grandcolas (1996) synonymized Archiblattidae with Blattidae. Recently Roth (2003) consented to this placement according to the male and female subgenital plates of three genera (*Archiblatta*, *Catara*, *Protagonista*) out of the four, but listed *Eroblatta* under Blattinae. From then on, the subfamily Archiblattinae was accepted as comprised of 3 genera (*Archiblatta*, *Catara* and *Protagonista*).

The genus *Protagonista* was established by Shelford (1908). He described *Protagonista lugubris* from the Manson Mountains, Tonkin (i.e. northern Vietnam) without any description of the male genitalia and designated it as the type species of *Protagonista*. Later Hanitsch (1923, 1925, 1929, 1931) described another four species belonging to the genus *Protagonista* from Southeast Asia: *Protagonista pertristis*, *Protagonista fusca*, *Protagonista aterrima* and *Protagonista laeta*. But Bruijning (1948) synonymized these 4 species with *Protagonista lugubris* Shelford, 1908 according to the difference in the depth of color of the tegmina and the whole body, which has less taxonomic value. Until now, the genus *Protagonista* was only reported from Vietnam, Malaysia, Indonesia and Singapore.

Previous studies of the subfamily Archiblattinae are fairly limited. In this paper, we report one known species *Protagonista lugubris* newly discovered from China, distributed in Hainan and Guangxi, of which the male and female genitalia are described in detail for the first time. A key to all species of *Protagonista* and the related genus *Eroblatta* is given. The taxonomic status of this subfamily and the genus *Eroblatta*, as well as the validity of the name Archiblattinae, are discussed. We also deal with the synonymy of *Protagonista lugubris* based on the examination of holotypes, geographical distribution and original descriptions.

## Material and methods

The terminology for the body, male and female genitalia used in this paper mainly follows McKittrick (1964) and Roth (2003). Terminology of veins follows Haas and Kukalová-Peck (2001) with modification by Li and Wang (2015). The specimens are deposited in the College of Plant Protection, Southwest University, Beibei, Chongqing, China (SWU), unless otherwise noted. Measurements are based on specimens examined. Widths of pronota and tegmina are based on their widest portion. The genital segments of the examined specimens were macerated in 10% NaOH and observed in glycerin jelly using a Motic K400 stereomicroscope. All drawings were made with the aid of a Motic K400 stereomicroscope. All specimens deposited in SWU were photographed using a digital camera (Canon EOS 50D) coupled with a macro lens (Canon EF 100mm f/2.8 USM). The photographs were processed in Helicon Focus software.

The terms of veins (abbreviations given in parentheses) are: subcosta (*Sc*), radius (*R*), radius anterior (*RA*), radius posterior (*RP*), media (*M*), cubitus anterior (*CuA*), cubitus posterior (*CuP*), anal (*A*), anal anterior (*AA*), anal posterior (*AP*). The terms of female genitalia (abbreviations given in parentheses) are: paraprocts (*pp.*), anterior arch (*a.a.*), paratergites (*pt.*), first valve (*v.I*), second valve (*v.II*), third valve (*v.III*), laterosternite IX (*ltst.IX*), basivalvula (*bsv.*), laterosternal shelf (*ltst.sh*), common oviduct opening (*c.o.o.*), spermathecal opening (*sp.o.*), and vestibular sclerite (*vst.s.*).

The terminology of Roth (2003) is used in describing the spines (armament) on the antero-ventral margin of the front femur, where type A refers to a row of stout or “heavy” spines which decrease gradually in size distad, terminating in two or three large spines, rarely up to five large terminal spines. The number of stout terminal spines are indicated by subscripts so that one or two terminal spines are Type A_1_ or A_2_.

The standard barcoding sequences of the mitochondrial COI gene (658 bp) of *Protagonista lugubris* from Hainan and Guangxi are approved, which are deposited in GenBank under the accession numbers KU511283, KU511284, KU511285 and KU511286.

## Taxonomy

### Subfamily Archiblattinae

Taxon classificationAnimaliaBlattodeaBlattidae

Kirby, 1904 (1868) new record from China

Planeticidae Walker, 1868: 25; Walker 1869: 121; Princis 1965: 386. Type genus: Planetica Saussure, 1863.
Archiblattinae Kirby, 1904: 148, as a substitute name based on the synonymy of Planetica with Archiblatta; Princis 1965: 386; Roth 2003: 33. Type genus: Archiblatta Snellen van Vollenhoven, 1862.
Archiblattidae : Handlirsch 1930: 836; Princis 1960: 439; Princis 1965: 386.

### Genus Protagonista

Taxon classificationAnimaliaBlattodeaBlattidae

Shelford, 1908 new record from China

Protagonista Shelford, 1908: 158; Shelford 1910: 22; Hanitsch 1923: 443; Bruijning 1948: 117; Princis 1965: 388; Grandcolas 1996: 520; Roth 2003: 33. Type species: Protagonista lugubris Shelford, 1908.

#### Generic diagnosis.

The genus *Protagonista* is remarkable on account of the shape of the pronotum (as long as broad, quadrangular, with rounded angles, sides not deflexed), and the pubescence on its pronotum and tegmina (after Shelford 1908). The other three genera of Archiblattinae differ from it by the apterous female and the unarmed or weakly-armed femur (*Archiblatta* and *Catara*) or by the tibia having three rows of spines (*Eroblatta*).

#### Description.

**Figures 1–6.**
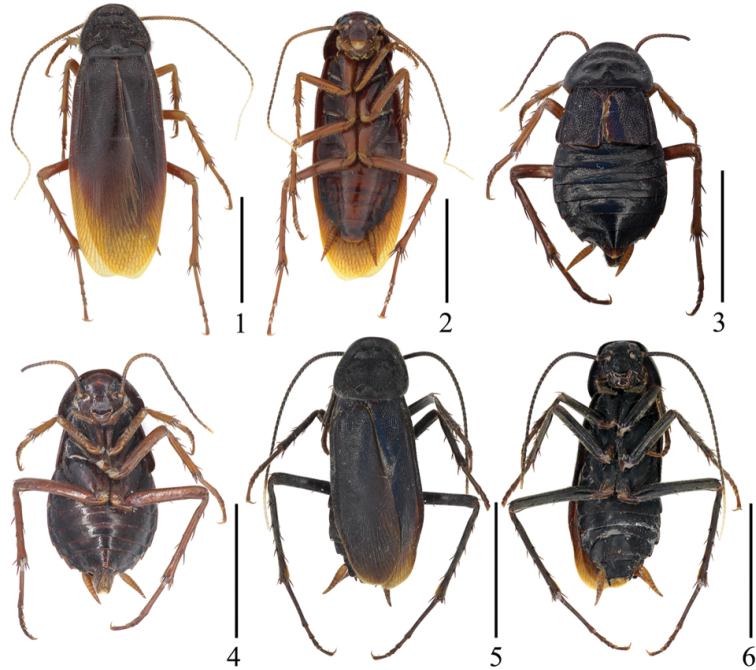
*Protagonista lugubris* Shelford. **1–2** Male from Hainan: **1** dorsal view **2** ventral view **3–4** Female from Hainan: **3** dorsal view **4** ventral view **5–6** Male from Guangxi: **5** dorsal view **6** ventral view. Scale bars: 1.0 cm.

Antennae slightly moniliform. Ocelli present. Pronotum as long as broad, quadrangular, with rounded angles, sides not deflexed and not covering vertex (Figs 1–6 appears to show it covering the vertex, however this is an artifact of the photo angle). Pronotum and tegmina with fine pubescence. Tegmina and hind wings fully developed in the male, exceeding the apex of the abdomen. Tegmina short and truncated in the female, hind wing vestigial to a small lobe. Styli present and cerci moderate. Legs slender; front femora Type A_2_; hind tibia with 2 rows of spines along outer margin; hind metatarsus very long, considerably exceeding the remaining joints in length; the tarsal pulvilli present on the proximal four tarsomeres; arolia minute.

#### Distribution.

Vietnam; Malaysia (Malacca State); Sumatra; Borneo; China (new record; Hainan, Guangxi).

### Protagonista lugubris

Taxon classificationAnimaliaBlattodeaBlattidae

Shelford, 1908 new record from China

Figs 1–6 , 7–12 , 13–22 , 23–27 , 28–33 , 34–43 , 45 , 48–49

Protagonista lugubris Shelford, 1908: 158; Shelford 1910: 22; Hanitsch 1927: 40; Hanitsch 1929: 17; Bruijning 1948: 117.

#### Description.

**Male.** Body slender, dark brown to black (Figs 1–2, 5–6, 49). Eyes black, ocelli yellowish white. Vertex and face dark brown to black. Clypeus yellowish or dark brown and the base of labrum pale, labial palpi and maxillary palpomeres brown. Antennae brown or black, apical joints creamy-white. Pronotum and tegmina dark brown or black and apex of tegmina brownish yellow or brown. Legs brown or black. Abdomen reddish brown with dark brown margins or uniformly black. Cerci brown or yellowish brown.

**Figures 7–12.**
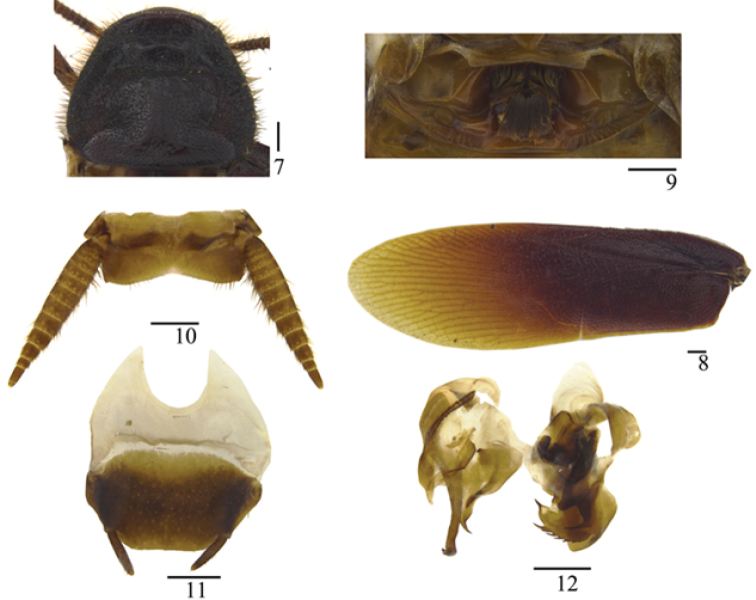
*Protagonista lugubris* Shelford, male from Hainan: **7** pronotum **8** tegmen **9** abdominal tergum 1, dorsal view **10** supra-anal plate and paraprocts, ventral view **11** subgenital plate, dorsal view **12** left phallomere and right phallomere. Scale bars: 1.0 mm.

**Figures 13–22.**
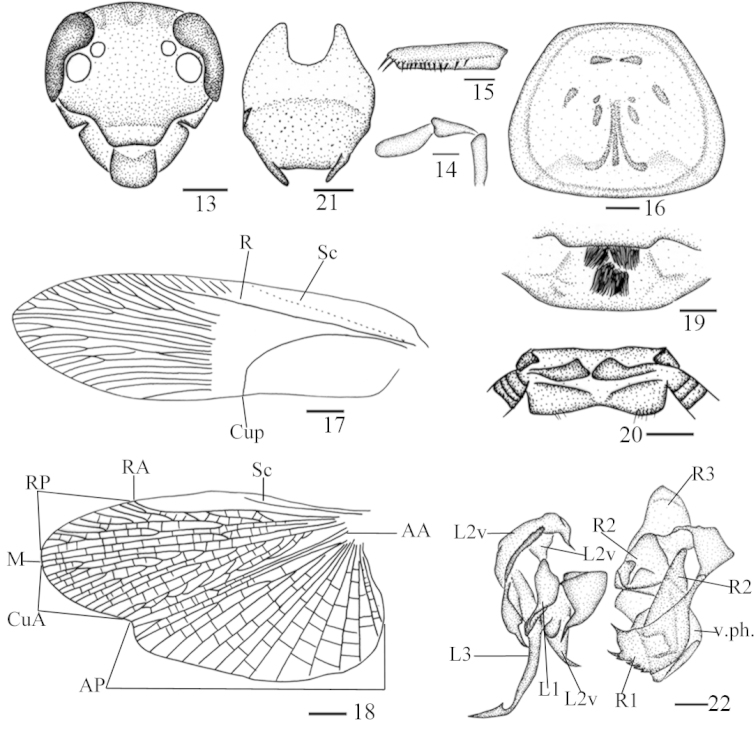
*Protagonista lugubris* Shelford, male from Hainan: **13** head, frontal view **14** maxillary palps 3–5 **15** front femur **16** pronotum **17** tegmen **18** hind wing **19** abdominal tergum 1, dorsal view **20** supra-anal plate and paraprocts, ventral view **21** subgenital plate, dorsal view **22** left phallomere and right phallomere. Scale bars: 1.0 mm (**13, 15–16, 19–21**), 0.5 mm (**14, 22**), 2.0 mm (**17–18**).

Head with vertex punctate, with three smooth longitudinal stripes (Figs 13, 34). Eyes reniform and closer together than antennal sockets. Ocelli elliptical with distinct border. Face punctate, with some smooth interspaces and lines (Figs 13, 34). Antennae with numerous bristles, shorter than the body. Pronotum elongate, trapezoidal, with punctations and pubescence, margins thickened and raised, with three smooth longitudinal impressions and some smooth interspaces, disk not flat (Figs 7, 16, 28, 37). Both tegmina and hind wings fully developed, extending beyond the end of abdomen. Tegmina narrow with scattered erect pubescence; basal half sclerotized (Figs 8, 29), veins indistinct other than *Sc*, *R* and *CuP*; *CuP* ending at the middle of the hind margin, *A* almost invisible (Figs 17, 38). Hind wing with indistinct *Sc*, *M* bifurcated; *CuA* with 9 branches, of which four branch again (Figs 18, 39). Legs slender with dense pubescence. Tarsal claws symmetrical and unspecialized. First abdominal tergum specialized, with dense setae medially (Figs 9, 19, 30, 40).

Supra-anal plate in ventral view symmetrical, hind margin convex with a weak medial indentation, two paraprocts large and nearly symmetrical (Figs 10, 20). Cerci conical and segmented (Figs 10, 31). Subgenital plate in dorsal view nearly symmetrical; styli modest, cylindroid (Figs 11, 21, 32, 42). Left phallomere consisting of three parts: *L1*, *L2* and *L3*; sclerite *L1* folded with a narrow and fingerlike terminus; sclerite *L2* large, flat, and folded over posteriorly so that *L2v* lies on the ventral surface of the phallomere and *L2d* on the dorsal, sclerite *L2d* with a rough and curved margin, terminus acute, sclerite *L2v* with two acute ends posteriorly, of which (in dorsal view) the left one tapers towards the right and the other towards the left; sclerite *L3* forming an elongate hook of which the curved part has a small spinous protuberance (Figs 12, 22, 33, 43) that is inconspicuous due to the observation angle. Right phallomere consisting of three parts: *R1*, *R2* and *R3*; sclerite *R1* expanding downward towards the left and with a serrate edge; *R2*, hook-like, expanding towards the left; the basal sclerite of *R3* broad and slightly curved, joining with *R2*. The ventral phallomere (*v.ph.*) under the right phallomere, flat, posteriorly rounded, with a more or less sclerotized ventral surface (Figs 12, 22, 33, 43).

**Female.** Body black (Figs 3–4, 48). Eyes, ocelli and antennae similar to those of male. Vertex and face reddish brown. Labial palpi and maxillary palpomeres brown. Pronotum black. Abdominal terga black, but with the last segment brown. Abdominal sterna black and center reddish brown. Legs and cerci brown.

Vertex exposed, with 3 longitudinal shining stripes. Face punctuated. Tegmina short, just exceeding the metanotum, with punctures and scattered erect pubescence, heavily sclerotized with metallic shine. Hind wings much reduced. Legs slender, front femur Type A_2_. Each hind tibia with 2 rows of spines along outer margin. Hind metatarsus exceeding the remaining joints in length.

**Figures 23–27.**
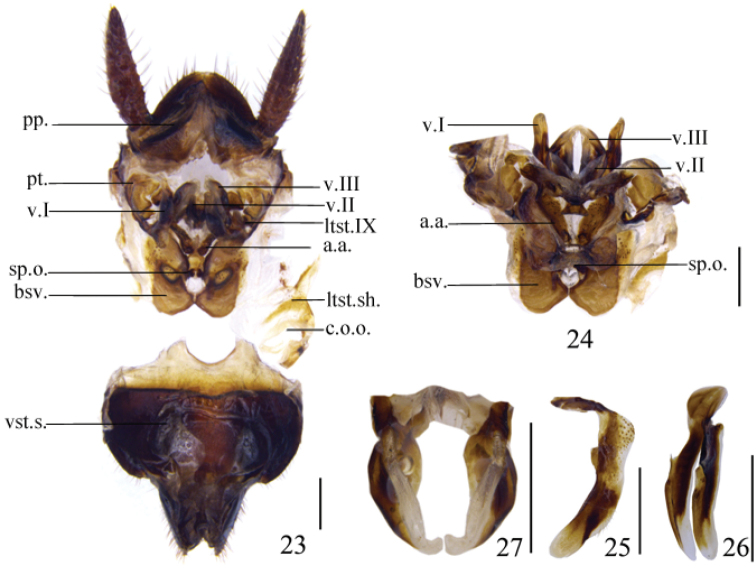
*Protagonista lugubris* Shelford, female from Hainan, female genitalia: **23** posterior view and dorsal view of subgenital plate **24** valves and accessory sclerites, dorsal view **25** first valve, ventral view **26** second valve, ventral view **27** third valve, ventral view. Scale bars = 1.0 mm.

Supra-anal plate nearly symmetrical, roof shaped, the hind margin nearly blunt and round; paraprocts (*pp.*) broad and similar (Fig. 23). The juncture between the spermatheca plate and the anterior arch (*a.a.*) membranous and somewhat extensible (Fig. 23). First valve (*v.I*) falciform, sclerotized, with slender base and weakly sclerotized terminus (Figs 23, 24, 25); laterosternite IX (*ltst.IX*) large, fused to paratergites (*pt.*); paratergites (*pt.*) slender (Fig. 23). Second valve (*v.II*) small and slender, basally fused, connecting to third valve (*v.III*) by membrane (Figs 23, 24, 26). Third valve larger than second valve but smaller than first valve, with weakly sclerotized and curved apex, basal portion fused and slightly raised (Figs 23, 24, 27). Anterior arch (*a.a.*) claviform, with tapering terminus (Fig. 23). Well developed basivalvula (*bsv.*) strongly sclerotized, fused with the anterolateral deflections of the spermatheca plate. Laterosternal shelf (*ltst.sh.*) flat, divided by common oviduct opening (*c.o.o.*). Subgenital plate in dorsal view symmetrical (Fig. 23).

**Nymph.** Body color, characters of pronotum and antennae similar to those of adults. Legs light brown. Cerci reddish brown.

**Infraspecific variation.** The individual differences in morphological characters mainly involve: 1) the number and shape of smooth areas of pronotum (Figs 7, 16, 28, 37); 2) the dentate tine close to the largest tine of the serrated edge of sclerite *R1* sclerotized (Figs 12, 22) or not sclerotized (only one case, Figs 33, 43); 3) body color (Figs 1–2, 5–6). We provide pictures for detailed comparison (one male from Hainan, one male from Guangxi) illustrating the appearance of individual differences (Figs 1–2, 5–6, 7–12, 13–22, 28–33, 34–43). These infraspecific variations cannot separate the populations from each other into different species and the key morphological characters strongly suggest they are conspecific. However, their COI genes show a great genetic divergence among them: the standard barcoding sequence of one (Baisha) of the three Hainan populations has a distance of 3.0% and 3.1% from the other two (Wuzhishan, Baoting) respectively, and it is far distant (4.6%) from the Guangxi population, which in turn is very distinct from the two remaining Hainan populations (Wuzhishan, 6.1%; Baoting, 6.2%).

**Male measurements (mm).** Body length: 17.5–21.0. Total length including tegmen: 19.0–24.5 . Pronotum length × width: 4.9–5.5 × 5.5–6.5. Tegmen length × width: 15.0–20.0 × 5.0–6.5.

**Female measurements (mm).** Body length: 19.0–20.0. Pronotum length × width: 5.5–6.0 × 6.0–6.5. Tegmen length × width: 4.7–5.0 ×4.7–5.0.

**Figures 28–33.**
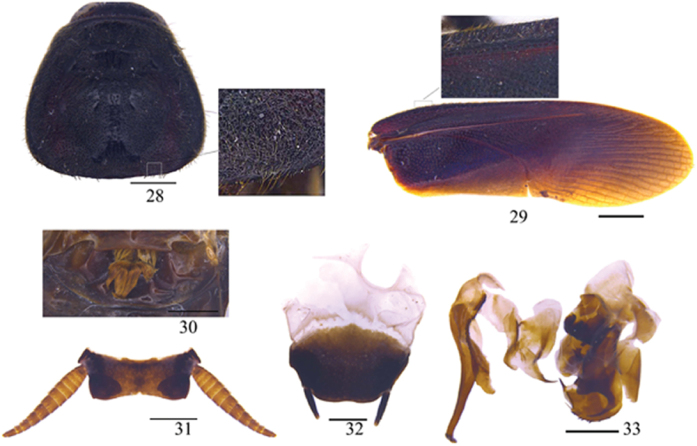
*Protagonista lugubris* Shelford, male from Guangxi: **28** pronotum **29** tegmen **30** abdominal tergum 1, dorsal view **31** supra-anal plate and paraprocts broken, ventral view **32** subgenital plate, dorsal view **33** left phallomere and right phallomere. Scale bars: 1.0 mm (**28, 30–33**), 2.0 mm (**29**).

**Figures 34–43.**
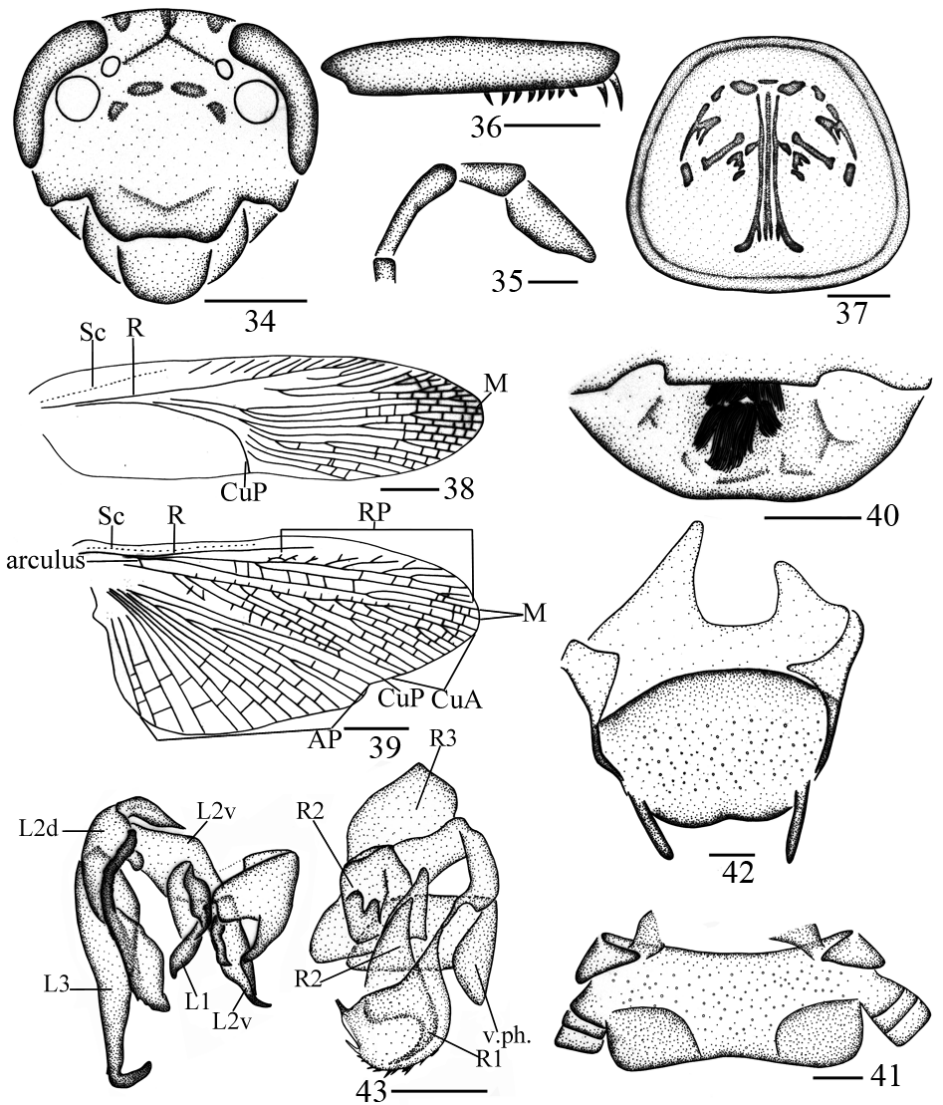
*Protagonista lugubris* Shelford, male from Guangxi: **34** head, frontal view **35** maxillary palps 3–5 **36** front femur **37** pronotum **38** tegmen **39** hind wing **40** abdominal tergum 1, dorsal view **41** supra-anal plate and paraprocts broken, ventral view **42** subgenital plate, dorsal view **43** left phallomere and right phallomere. Scale bars: 1.0 mm (**34, 36–37, 40**), 0.5 mm (**35, 41–42**), 2.0 mm (**38–39**).

**Figures 44–47.**
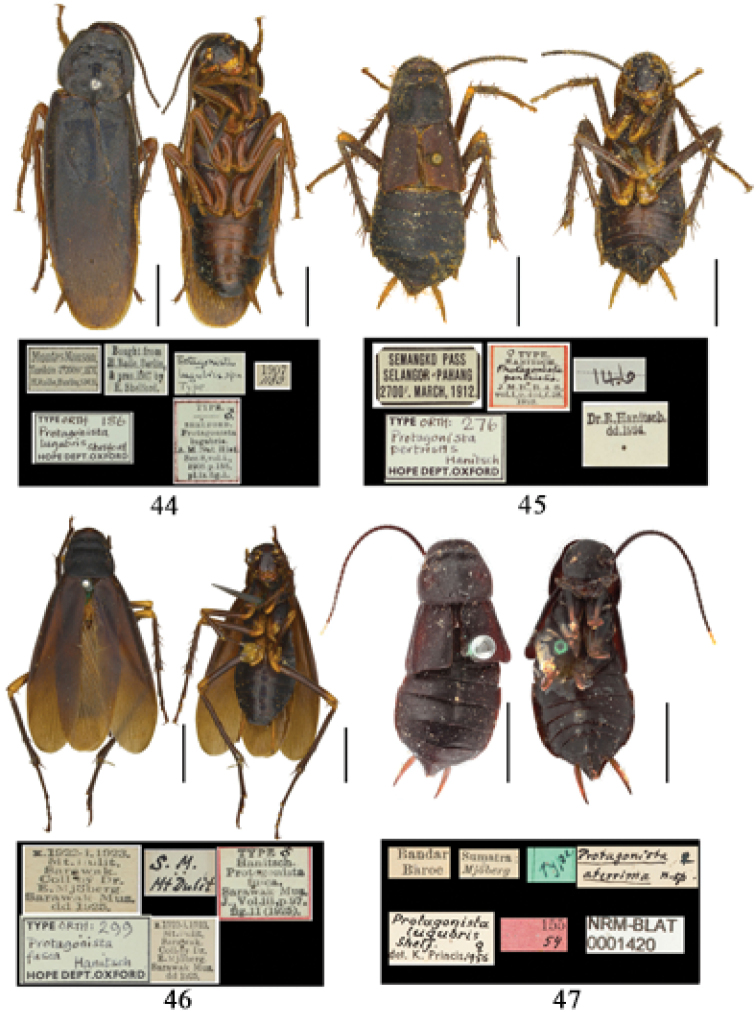
*Protagonista* species, holotypes and labels. **44** *Protagonista lugubris*, male **45** *Protagonista pertristis* stat. rev., female **46** *Protagonista fusca*, now synonym of *Protagonista pertristis*, male **47** *Protagonista aterrima*, now synonym of *Protagonista pertristis*, female. Scale bars: 5.0 mm.

**Figures 48–49.**
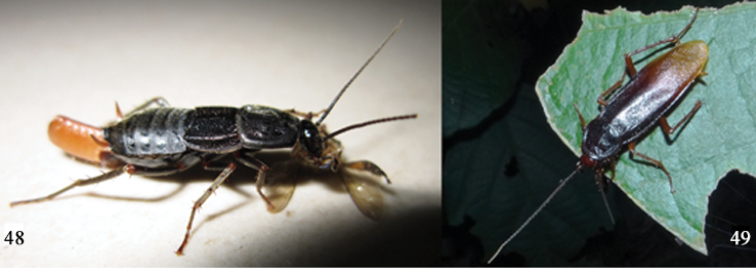
**48** *Protagonista lugubris* Shelford, adult female from Longtan Park in Guiping, Guangxi, 2011.VIII.4, eating a wasp body near a light trap **49** *Protagonista lugubris* Shelford, adult male from Diaoluo Mt. in Lingshui, Hainan. Both photographed by XinRan Li (= Conlin McCat).

**Figure 50.**
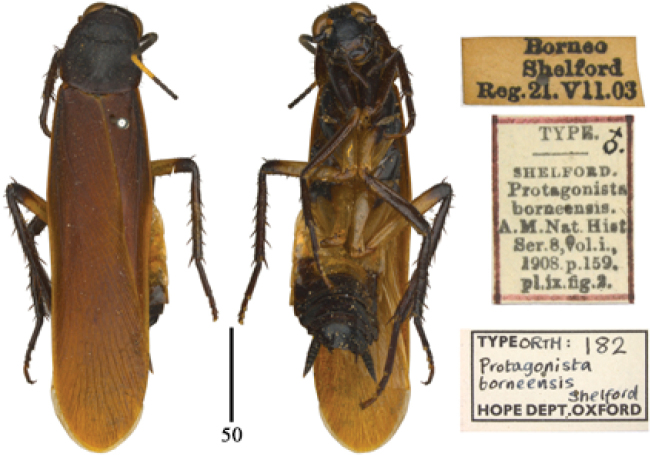
*Eroblatta borneensis* (Shelford 1908), holotype and labels. Scale bar: 5.0 mm.

#### Material examined.

One male and one nymph, China: Hainan Prov., Baisha County, Yinggeling Natural Reserve, Nankai Station, in rotten wood, 21 April 2015, coll. Xinran Li (=Conlin McCat) and Zhiwei Qiu; one male, China: Hainan Prov., Mt. Diaoluoshan, 275m, 18°40.080'N, 109°53.998'E, 25 May 2014, coll. Shunhua Gui and Xinran Li (=Conlin McCat); one male, China: Hainan Prov., Baoting County, Maogan Township, 11–12 April 2015, coll. Qikun Bai; one female, China: Hainan Prov., Mt. Wuzhishan, 795m, 18–21 May 2014, coll. Shunhua Gui, Xinran Li (=Conlin McCat) and Jianyue Qiu; one male, China: Guangxi Aut. Reg., Guiping City, Longtan Park, 386 m, 23°31.140'N, 109°59. 510'E, 31 May–2 June 2014, coll. Shunhua Gui and Xinran Li (=Conlin McCat); one male and two females, China: Guangxi Aut. Reg., Fangchenggang City, Shangsi County, Shiwandashan Forest Park, 296 m, 28 June 2015, coll. Lu Qiu and Qikun Bai.

#### Distribution.

China (new recored; Hainan, Guangxi); Vietnam.

#### Habitat.

The adult *Protagonista lugubris* were observed in shrubs at night by the collectors who also found the nymphs and adults in rotten wood. Their rugged pronotum with thickened and raised margins, which resembles that of Cryptocercinae and Panesthiinae is conducive to moving about in rotten wood. However, if *Protagonista* utilizes the wood tunneled by other organisms or if they bore the wood themselves is yet to be seen. Additionally, wood feeding has not been observed but is still a possibility in *Protagonista* and the other morphologically similar Archiblattinae.

## Discussion

**Validity of the name Archiblattinae.** As explained in the introduction, the scientific names Archiblattinae and Planeticinae are synonyms. According the *Principle of Priority* in ICZN, Archiblattinae should be abandoned and the earlier name Planeticinae is valid although its type genus is no longer valid. But the name Planeticinae/–idae has been ignored for a long time and the substitute name Archiblattinae/–idae is in prevailing usage since Princis’s (1965) catalogue; therefore the priority of the latter should be maintained based on the ICZN rule 40.2.

**Taxonomic status of subfamily Archiblattinae and its genera.** The subfamily Archiblattinae has a controversial taxonomic history since it was established. Although Kirby (1904) erected the subfamily Archiblattinae based on the genus *Archiblatta*, Shelford (1910), Hanitsch (1915) and Bruijning (1948) placed *Archiblatta* in Blattinae. Subsequent authors also have different suggestions on the taxonomic status of family Archiblattidae. Princis (1965) listed it as a family, but Grandcolas (1996) synonymized Archiblattidae with Blattidae and assigned *Archiblatta* and *Catara* to Blattidae. Roth (2003) suggested that *Archiblatta, Catara* and “?*Protagonista*” should be in their own subfamily because of the absence or greatly reduced femoral armament and reserved the subfamily Archiblattinae (Fam. Blattidae). Inward et al (2007) and Legendre et al (2015) found the subfamily Archiblattinae (*Archiblatta*) and Blattinae to be respectively monophyletic. Klass and Meier (2006) placed *Archiblatta* as sister to Polyzosteriinae + Blattinae. Djernæs et al. (2015) indicated that the structuring of Blattidae into the subfamily Polyzosteriinae (*Drymaplaneta*, *Eurycotis*), Archiblattinae (*Archiblatta*), and Blattinae (*Periplaneta*, *Deropeltis*) may be artificial since Archiblattinae were placed within Blattinae. In spite of the few studies on the Archiblattinae, our knowledge about the genera other than *Archiblatta* is still so lacking that it is reasonable to question their classification in Archiblattinae. There is uncertainty regarding the taxonomic status of *Catara*, *Protagonista*, and *Eroblatta*. When comparing the male genitalia of *Protagonista lugubris* with those of *Archiblatta hoeveni* (illustrated by Klass 1997) and other species (e.g. *Periplaneta americana*, *Periplaneta brunnea*, *Periplaneta ceylonica*, *Blatta orientalis*, *Neostylopyga rhombifolia*, *Melanozosteria nitida*) in the subfamilies Blattinae and Polyzosteriinae, we find that the male genitalia of Archiblattinae and Blattinae are closer to each other than to those of Polyzosteriinae. Furthermore, *Protagonista* and *Archiblatta* are more similar to each other than they are to the genera of Blattinae. However, we failed to find independent, distinct morphological features separating the two taxa; thus the male genital differences between them might not be adequate as diagnostic characters in subfamily-group taxonomy. Our observations coincide, in a phylogenic sense, with Djernæs et al. (2015). However, Archiblattinae is easily distinguished from other blattid cockroaches by the special pronotum (hardened and rugose, sides thickened and not deflexed) and the special tibia which are extraordinarily cylindrical with sparse spines. Additionally, the cladistic results themselves are still in dispute. Yet we should not simply rely on the cladistic results solely to alter the classification, even if a widely accepted cladistic conclusion is demonstrated in the future. Therefore we propose to retain the validity of the subfamily Archiblattinae and the arrangement of the 4 genera mentioned above before a comprehensive taxonomic and phylogenetic study has been conducted. If done, this future study should on one hand confirm whether the subfamily Archiblattinae is monophyletic, and should on the other hand discern the relationships among the 4 genera and give an acceptable arrangement of them.

**Synonymy of *Protagonista lugubris*.** Hanitsch (1932) synonymized *Protagonista aterrima* Hanitsch, 1929 with *Protagonista fusca* Hanitsch, 1925. Bruijning (1948) synonymized 4 species (*Protagonista aterrima*, *Protagonista fusca*, *Protagonista pertristis* Hanitsch, 1923 (Figs 44–47) and *Protagonista laeta* Hanitsch, 1931) with *Protagonista lugubris* Shelford, 1908, as he considered a slightly differing coloration not an important specific character that is coincident with Hanitsch (1932). We also consider the four nominal species *Protagonista aterrima*, *Protagonista fusca*, *Protagonista pertristis* and *Protagonista laeta* to be the same species, but separate from *Protagonista lugubris* Shelford, 1908. We base this on evidence from holotypes, original descriptions and geographical distribution. They are all from a limited region (Sunda Shelf) far away from the localities of *Protagonista lugubris* (Northern Vietnam and South China). We agree with Hanitsch’s (1932) synonymy based on the holotypes: the distal half of coxae and the base of femora of *Protagonista aterrima* are light testaceous, as is *Protagonista fusca*. Meanwhile we indicate that Bruijning’s (1948) viewpoint should be revised: *Protagonista aterrima*, *Protagonista fusca*, *Protagonista pertristis* and *Protagonista laeta* are identical indeed, but they are not synonyms of *Protagonista lugubris*. Therefore *Protagonista aterrima*, *Protagonista fusca* and *Protagonista laeta* are the junior synonyms of *Protagonista pertristis*. *Protagonista pertristis* is distinguished from *Protagonista lugubris* by the following characters: 1) coxae of all the legs with the distal half and the base of femora are orange yellow on *Protagonista pertristis* and *Protagonista fusca*, but all the legs of *Protagonista lugubris* are uniformly brown (Note: The character of difference in color depth of tegmina and the whole body has less taxonomic value, in accordance with Hanitsch (1932) and Bruijing (1948). However, the distinct coloration of part of the body (such as this case) should be the criteria for species differentiation.); 2) cerci of *Protagonista pertristis* and *Protagonista fusca* slender, yellow, but those of *Protagonista lugubris* thicker and shorter, brown. In conclusion, the genus *Protagonista* is comprised of 2 species, *Protagonista lugubris* Shelford, 1908 and *Protagonista pertristis* Hanitsch, 1923, the latter with three junior synonyms: *Protagonista aterrima*, *Protagonista laeta* and *Protagonista fusca*.

**Taxonomic status of the genus *Eroblatta*.** The genus *Eroblatta* was erected based on *Protagonista borneensis* Shelford, 1908 by Shelford (1910). The genera *Eroblatta* and *Protagonista* differed from each other in the spines on outer margin of tibiae according to his original description. *Eroblatta* has 3 rows of spines along outer margin of tibia, whereas there are only 2 rows in *Protagonista*. *Eroblatta* was also placed in Archiblattidae by Princis (1965) but Roth (2003) listed it under Blattinae without any explanation; within the period between these two publications (i.e. Princis 1965 and Roth 2003), works with respect to *Eroblatta* were absent, the change on its taxonomic arrangement is deemed to be simply an inadvertent error. After examining the holotype (Fig. 50) and according to original description, it is seen clearly that *Eroblatta borneensis* has the typical archiblattid pronotum (hardened and rugose with sides thickened and not deflexed) and the special tibia, which are extraordinarily cylindrical with sparse spines. Therefore *Eroblatta* is exactly, for the time being, a member of Archiblattinae.

### Checklist of the synonyms of the species of *Protagonista* and *Eroblatta*

**Table:** Checklist of the synonyms of the species of *Protagonista* and *Eroblatta*

***Protagonista***	
*Protagonista lugubris* Shelford, 1908	Vietnam (Tonkin, type locality), South China
*Protagonista pertristis* Hanitsch, 1923, **stat. rev.**	Sunda Shelf including Malay Peninsula (type locality)
syn. *Protagonista fusca* Hanitsch, 1925	Sarawak
syn. *Protagonista aterrima* Hanitsch, 1929	Sumatra
syn. *Protagonista laeta* Hanitsch, 1931	Singapore
***Eroblatta***	
*Eroblatta borneensis* Shelford, 1908	Borneo (Sarawak, type locality)

### Key to the species of *Protagonista* and *Eroblatta*

**Table:** 

1	Tibia with 3 rows of spines along outer margin	***Eroblatta borneensis***
–	Tibia with 2 rows of spines along outer margin	(*Protagonista*) **2**
2	Coxae of all legs with the distal half and the base of femora orange yellow	***Protagonista pertristis***
–	All legs uniform in colour	***Protagonista lugubris***

## Acknowledgements

We are sincerely grateful to reviewers Marie Djernæs, Michael Kotyl and Sonia Lopes for providing helpful comments on this paper. We also express our thanks to Amoret Spooner (Oxford University Museum of Natural History, Hope Entomological Collections) and Gunvi Lindberg (Naturhistoriska Riksmuseet) for providing photographs of the holotypes. We thank colleagues Lu Qiu, Qikun Bai, Xinran Li (= Conlin McCat), Zhiwei Qiu, Shunhua Gui and Jianyue Qiu for collecting specimens, among them Xinran Li also took the photos of *Protagonista lugubris* in the wild.We also thank Prof. John Richard Schrock (Department of Biological Sciences, Emporia State University, USA) for revising this manuscript.

This project was supported by a Program of Ministry of Science and Technology of the People’s Republic of China (2015FY210300), the National Natural Sciences Foundation of China (31472026, 31093430 and 31493021) and The Fundamental Research Funds for the Central Universities, China (XDJK2013B013, 2362015XK04).

## Supplementary Material

XML Treatment for Archiblattinae

XML Treatment for Protagonista

XML Treatment for Protagonista lugubris
